# Characterization of the Probiotic Potential of Lactic Acid Bacteria Isolated from Kimchi, Yogurt, and Baby Feces in Hong Kong and Their Performance in Soymilk Fermentation

**DOI:** 10.3390/microorganisms9122544

**Published:** 2021-12-09

**Authors:** Haicui Wu, Tim-Fat Shum, Jiachi Chiou

**Affiliations:** 1 Department of Applied Biology and Chemical Technology, The Hong Kong Polytechnic University, Hung Hom, Kowloon, Hong Kong, China; wu.hc.wu@connect.polyu.hk (H.W.); tim-fat-perry.shum@connect.polyu.hk (T.-F.S.); 2 Research Institute for Future Food, The Hong Kong Polytechnic University, Hung Hom, Kowloon, Hong Kong, China

**Keywords:** LAB, soymilk, tolerant ability, fermentation characteristics

## Abstract

**Background:** There are several potential healthy or nutritional benefits from the use of lactic acid bacteria (LAB) in foods. This study aimed to characterize the LAB isolates from kimchi, yogurt, and baby feces in the Hong Kong area and evaluate their performance in fermented soymilk, which allowed us to assess their potential use in future experiments. **Methods:** General characteristics including tolerance to acid, NaCl, bile salts and phenol, antimicrobial activity to various pathogens, and adhesive ability to Caco-2 cells were evaluated using 18 LAB in this study. To further demonstrate the influence of such isolates in soymilk fermentation, we measured viability by plating and noting changes in pH, amino acid content, aglyconic isoflavones content and antioxidant capacities in vitro, such as scavenging ability, and iron chelating ability. **Results:** In this study, various LAB isolates belonging to *Lactobacillus* *rhamnosus*, *Lactobacillus sakei*, *Lactiplantibacillus plantarum*, *and* *Leuconostoc* *mesenteroides* isolated in Hong Kong were evaluated. *L. plantarum* isolates R7, AC12, and AC14.1, and *L. rhamnosus* AC1 showed higher tolerance to acid, NaCl, bile salts, and phenol as compared to the other isolates tested. *L. plantarum* isolates AC12, AC13 and AC14.1, and *L. rhamnosus* AC1 harbored strong antimicrobial activity. *L. plantarum* isolates R7, AC12, AC13 and AC14.1, and *L. paracasei* isolates R6 and R8 showed higher adhesive ability than the other tested isolates. In soymilk, the viable numbers of *L. paracasei* R5, *L. plantarum* R7, *L. rhamnosus* AC1, *L. sakei* AC2, and *Leu. mesenteroides* AC5 were much higher than the other tested isolates after 48 h of fermentation. The pH value measuring the lactic acid level in soymilk fermented by *L. plantarum* AC14.1 was the lowest in comparison to those in soymilk fermented by other isolates. In addition, the levels of free amino acids and isoflavones in the aglycone forms of *L. rhamnosus* AC1-fermented soymilk were the highest. *L. rhamnosus* AC1-fermented soymilk also showed the highest antioxidant potential, including DPPH scavenging ability and iron chelating ability. **Conclusions:** In general, *L. plantarum* isolates R7 and AC14.1 and *L. rhamnosus* AC1 exhibited higher tolerance to challenging conditions as compared to the other isolates. Moreover, *L. rhamnosus* AC1 exhibited superior performance in soymilk fermentation and potential as a starter and probiotic culture.

## 1. Introduction

Probiotics are defined as “live microorganisms that, when administered in adequate amounts, confer a health benefit on the host” by the Food and Agriculture Organization of the United Nation and the World Health Organization (FAO/WHO) [1]. Lactic acid bacteria (LAB), one of the major groups of probiotic microorganisms, are Gram-positive, facultative anaerobic bacteria, and produce lactic acid as the main fermentation product when hydrolyzing carbohydrates [2]. Most of the LAB are normal flora of the human gastrointestinal (GI) tract and confer varied beneficial effects in human beings, such as immunomodulation, antiallergic effects, antifungal actions, preservation of food, and protection against colon cancer [3,4,5,6,7,8]. Numerous studies have shown there is an increasing quantity, availability, digestibility, and assimilability of nutrients in foods fermented by the *Lactobacillus* species [9]. Lactose intolerance can be reduced by regularly consuming the fermented dairy products due to the presence of lactase produced by LAB in these products [10]. The use of LAB-containing food such as yogurt could improve diarrheal disease, especially in children [11].

The International Scientific Association for Probiotics and Prebiotics (ISAPP) developed a new definition for fermented foods and beverages as “food made through desired microbial growth and enzymatic conversions of food components” in September 2019 [12]; however, the differences between fermented foods and probiotic foods need to be clarified. The term “probiotic foods” should only be used when there is a demonstrated health benefit conferred by well-defined and characterized live microorganisms in the fermented foods and beverages. The definition of fermented foods includes foods and beverages with living microorganisms present (e.g., yoghurt, sour cream, kefir, and most cheeses) or absent (e.g., bread, heat-treated or pasteurized fermented products, wine, and coffee) at the time of consumption [12].

Soymilk, a popular drink in Asia, contains 3% proteins but no cholesterol or lactose. Supplementation of soymilk has been shown to exert a hypocholesterolemic effect in a normolipidemic population [13]. Hooper et al. further conducted a meta-analysis to report the effects of soy proteins and isoflavones to potentially reduce the risk of hormone-associated health disorders in pre- and post-menopausal women [14]. However, soymilk is less preferred by some consumers due to the beany flavor, flatulence factors, and high content of indigestible alpha-galactosyl oligosaccharides such as raffinose and stachyose in the soybean, which limit the consumption of soybeans as a raw food material [15]. LAB strains are commonly used in fermented products such as kimchi, silage, and dairy foods [16]. It has been reported that soymilk fermented by LAB can not only overcome the aforementioned limitations but increase the nutritional value of whole soybean [17]. Earlier studies have also shown the beneficial effects of soymilk for its antioxidant, hypolipidemic, and atherosclerosis-preventing activity on rodent models fed a high-fat diet [18,19].

Since LAB and their fermented products have a number of benefits for human health, it is relevant to identify isolates with potential as probiotic and starter cultures. This study aimed at identifying robust and competitive starter cultures for use in soymilk fermentation.

## 2. Methods

### 2.1. Bacterial Isolates and Culturing Conditions

Eighteen isolates belonging to eight groups, *L. acidophilus*, *L. paracasei*, *L. plantarum*, *L. delbrueckii subsp. Bulgaricus*, *L. rhamnosus*, *L. sakei*, and *Leu. mesenteroides* from varied samples, such as kimchi, yogurt, and baby feces were used in this study. All of the isolates summarized in Table 1 were cultured in MRS broth at 37 °C for 24 h anaerobically. Taxonomic identification in Table 1 was confirmed by sequencing of PCR-amplified 16S rDNA using the universal pair of primers 8F and 1512R.

### 2.2. Tolerance to Acid, NaCl, and Phenol

The isolates were grown in MRS broth at 37 °C for 14 h. An aliquot of 100 µL of each culture was transferred to the experimental MRS. Three experimental MRS were prepared. One set of MRS media was subjected to pH adjustment with 1 M of HCl to 2, 3, and 4 using pH meter (Thermo Scientific, Waltham, MA, USA, Orion Star A111.). A second set of experimental MRS broth was supplemented with sodium chloride (NaCl) to final concentrations of 3%, 6%, 9%, and 12% (*w*/*v*, 99.5% purity, M.W. 58.44). The third set of experimental MRS was supplemented with 0.2%, 0.4%, and 0.6% phenol (Sigma-Aldrich, St. Louis, USA, M.W. 94.11). Optical density at 600 nm was measured from each culture after incubation for 24 h. All of those isolates growing in regular MRS broth were regarded as control. Relative growth was determined by the ratio of OD_600_ of LAB cultures grown in the MRS supplemented with different concentration of acid, NaCl, and phenol to that in the regular MRS.

### 2.3. Tolerance to Bile Salts

The overnight cultures of LAB in the MRS broth were pelleted by centrifuge at 16,200× *g* for 1 min, re-suspended in PBS pH 7.4, and pelleted again. Final numbers of 8 × 10^7^ bacteria cells per ml were added into PBS solution with 0%, 0.3%, and 0.5% bile salts (*w*/*v*, Sigma-Aldrich, a mixture of 50% cholic acid sodium salt and 50% deoxycholic acid sodium salt, 0.1 g/mL of solubility in water), and incubated at 37 °C for 3 h. Serial dilution folds of bacteria suspensions in saline were conducted and plated in MRS agar in duplicate and incubated at 37 °C for 24 h anaerobically.

### 2.4. Adhesion Ability Assessment

A total of 2 × 10^4^ of Caco-2 cells was seeded into each well of the 24-well cell culture plates. Complete media (DMEM with 20% FBS) were changed every other day for two weeks until a monolayer of cells was formed. The LAB isolates were grown in MRS broth for 24 h. A total of 4 × 10^7^ of bacteria cells was added into each well of Caco-2 cells, followed by incubation in an anaerobic chamber at 37 °C for 2 h. After incubation, those cells were washed with PBS four times, and 100 µL of 1% Trition-X100 was used to lysate Caco-2 cells for 10 min. Optimal dilution of cell suspension was spread on the MRS agars, and they were incubated at 37 °C for 24 h anaerobically. This procedure was performed for each isolate in duplicate.

### 2.5. Antimicrobial Assessment

LAB isolates were grown in MRS broth for 24 h, and 2 µL of bacteria culture was applied as a single spot on the MRS plates containing 2% glucose and modified MRS plates (M-MRS) containing 0.2% glucose. Seven ml of semisoft BHI agar (0.7%) mixed with 10^5^ CFU/mL of the overnight-grown pathogenic bacteria including *Staphylococcus aureus*, *Acinetobacter baumannii*, *Salmonella typhimurium*, and *Pseudomonas aerugenosa* was overlaid on the MRS plates and M-MRS plates. Inhibition zones were recorded after 48 h of incubating at 37 °C. Each test was performed in duplicate.

### 2.6. Preparation of LAB-Fermented Soymilk

Soybeans were soaked in distilled water for 8 h, followed by blending and heating in 95 °C hot water for 2 h to make soymilk. A total of 3 × 10^6^ CFU/mL of each LAB isolate was used to ferment soymilk at 37 °C for different times.

### 2.7. Acidity, Proteolysis, and Viable Counts after Fermentation 

The changes in pH values of the soymilk fermented by the different LAB isolates were measured every 2 h using a pH meter. The proteolytic activity of LAB isolates was determined by measuring the production of free amino acid (FAA) using the ninhydrin assay according to the method described by Lekkas et al. [20]. Viable counts were enumerated at 0 and 48 h after fermentation, as previously described [21]. 

### 2.8. β-Glucosidase Activity Measurement in Fermented Soymilk

A total of 50 mL aliquots was taken aseptically from each sample of soymilk at different time points after fermentation to measure the β-glucosidase activity. The β-glucosidase activity was determined by measuring the rate of hydrolysis of ρ-nitrophenyl β-d-glucopyranoside (ρNPG), as described by Pyo et al. [22].

### 2.9. Determination of Isoflavones

The content of isoflavones was measured by HPLC. Fermented soymilk was dried to powder using a vacuum freeze drier for 2 days. The same amount of powder was dissolved to a final volume of 50 mL with methanol and sonicated for 30 min. After centrifuging for 10 min, the supernatant of samples was filtered through a 0.45 μm polytetrafluoroethylene filter, and isoflavones were quantified by HPLC, as described by Otieno [23]. Briefly, the isoflavone content was determined using a Keystone Scientific^®^ (Bellefone, PA, USA) ODS-C18 (250 mm × 4.6 mm internal diameter, 5 μm) reversed-phase column on a Hewlett Packard^®^ 1100 series HPLC (Agilent Technologies, Forest Hill, VIC, Australia) coupled with a UV detector. Genistein and Daidzein standards were used in this assay. Area of the peak represented the levels of aglycones.

### 2.10. Free Radical Scavenging Activity and Ferrous Ion-Chelating Activity

To determine the free radical scavenging capacity, 100 μL of each sample was added to 500 μL of 0.1 mM DPPH in 95% ethanol. The mixture was shaken and left for 60 min at room temperature in the dark, followed by reading the absorbance at 517 nm. Detailed information about the procedure is given by Kedare et al. in [24].

To measure the ferrous ion-chelating activity, 100 μL aliquots of each sample was added to 100 μL of 1% K_3_Fe(CN)_6_ and 100 μL of 0.2 M phosphate buffer. The mixture was shaken and incubated at 50 °C for 20 min. A total of 500 μL of 10% TCA was added to this mixture and centrifuged for 10 min. The supernatant was taken out to mix with distilled water and 0.1% FeCl_3_ (1:1:1), followed by reading the absorbance at 700 nm [25].

## 3. Results

### 3.1. Tolerance of LAB to Acid, NaCl, Bile Salt, and Phenol 

**Figure 1:**
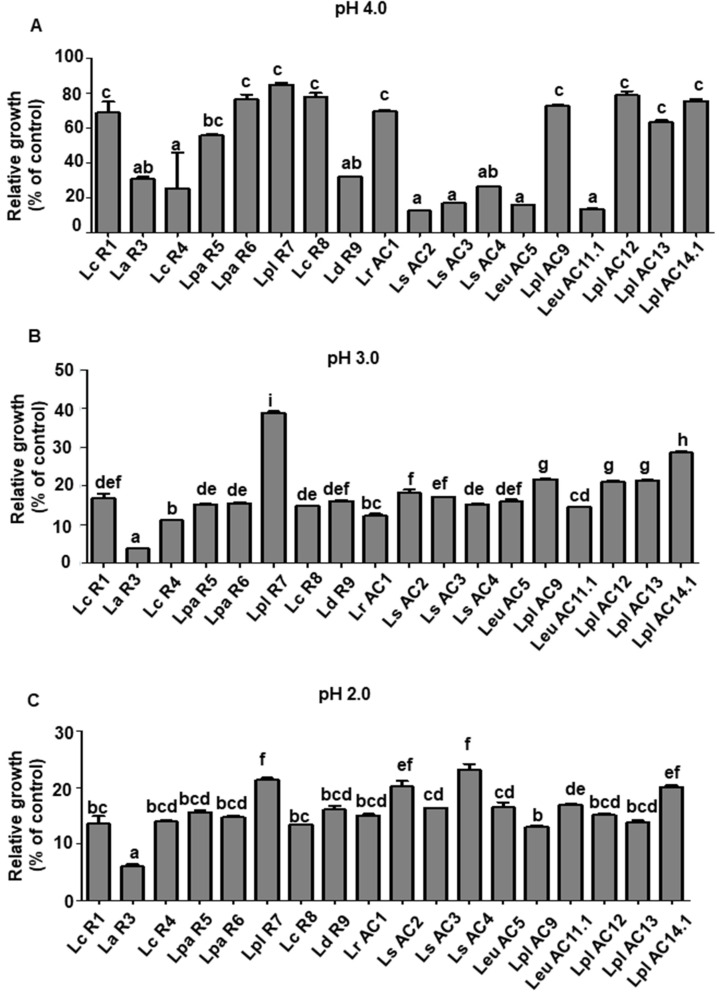
Relative growth of selected LAB isolates in the MRS broth with pH 4.0 (**A**), pH 3.0 (**B**), and pH 2.0 (**C**) at 24 h of incubation. Relative growth was determined by the ratio of OD_600_ of LAB cultures grown in the MRS adjusted to different pH to that in the regular MRS. Graph bars marked with different letters on top represent statistically significant results. The results were compared by one-way ANOVA and Turkey post hoc test; *p* < 0.05; *n* = 2. Lc, *L. casei*; La, *L. acidophilus*; Lpa, *L. paracasei*; Lpl, *L. plantarum*; Ld, *L. delbrueckii*; Lr, *L. rhamnosus*; Ls, *L. sakei*; Leu, *Leu. mesenteroides*.

The acidic pH in gastric juice is essential for human digestion. The growth of LAB isolates in MRS broth adjusted to different levels of pH was determined to evaluate their tolerance to acid. As shown in Figure 1A, the most tested LAB isolates grew well in the MRS at pH 4.0 except *L. sakei* *isolates* (AC2, AC3, and AC4), *Leu. mesenteroides* (AC5 and AC11.1), and *L. delbrueckii subsp. Bulgaricus* R9. Moreover, *L. rhamnosus* AC1, *L. plantarum* (R7, AC12, AC13, and AC14.1) and *L. paracasei* (R6 and R8) showed significantly higher tolerance to the medium with pH 4.0 compared with the other tested isolates (Figure 1A and Supplementary Figure S1). The *L. plantarum* R7 exhibited the highest tolerant ability, with almost 40% viability at pH 3.0, which is close to the pH of the human stomach (Figure 1B). All the isolates were not able to grow well in the MRS broth at pH 2.0 (Figure 1C and Supplementary Figure S1).

**Figure 2:**
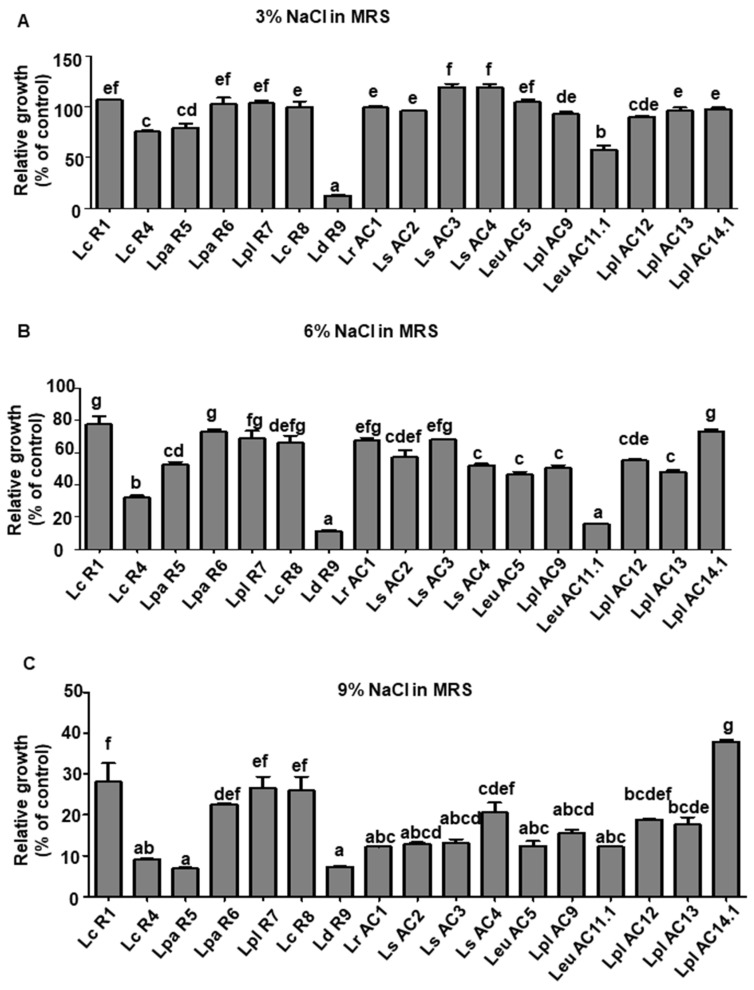
Relative growth of selected LAB isolates in the MRS broth supplemented with 3% (**A**), 6% (**B**), and 9% (**C**) NaCl for 24 h of incubation. Relative growth was determined by the ratio of OD_600_ of LAB cultures grown in the MRS supplemented with different concentrations of NaCl to that in the regular MRS. Graph bars marked with different letters on top represent statistically significant results. The results were compared by one-way ANOVA and Turkey post hoc test; *p* < 0.05; *n* = 2. Lc, *L. casei*; La, *L. acidophilus*; Lpa, *L. paracasei*; Lpl, *L. plantarum*; Ld, *L. delbrueckii*; Lr, *L. rhamnosus*; Ls, *L. sakei*; Leu, *Leu. mesenteroides*.

Similarly, the tolerance of LAB to NaCl was assessed by measuring their growth in the MRS broth supplemented with different concentrations of NaCl (Figure 2 and Supplementary Figure S2). Most of the isolates were able to grow in the MRS supplemented with a NaCl concentration of 3% except *L. delbrueckii subsp. Bulgaricus* R9 (Figure 2A). Other than *L. casei* R4, *L. delbrueckii subsp. Bulgaricus* R9, and *Leu. mesenteroides* AC11.1, the other 15 tested isolates maintained at least 50% growth in the MRS containing a NaCl concentration of 6% compared to that in the original MRS (Figure 2B). In addition, *L. plantarum* AC14.1 showed the highest tolerance, which was approximately 40% growth compared to original MRS and a NaCl concentration of 9% compared to other test isolates (Figure 2C and Supplementary Figure S2).

**Figure 3:**
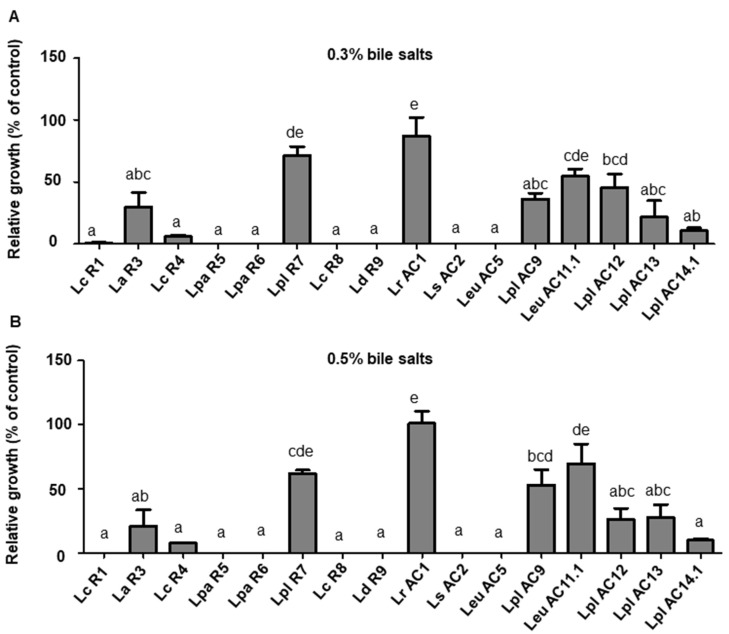
Relative growth of selected LAB isolates in the MRS plates supplemented with 0.3% (**A**) and 0.5% (**B**) bile salt after 24 h of incubation. Relative growth was determined by the colony-forming unit (CFU) of LAB grown in the MRS agars supplemented with different concentrations of bile salts to that in the regular MRS agar. Graph bars marked with different letters on top represent statistically significant results. The results were compared by one-way ANOVA and Turkey post hoc test; *p* < 0.05; *n* = 2. Lc, *L. casei*; La, *L. acidophilus*; Lpa, *L. paracasei*; Lpl, *L. plantarum*; Ld, *L. delbrueckii*; Lr, *L. rhamnosus*; Ls, *L. sakei*; Leu, *Leu. mesenteroides*.

Bile salts are important factors that facilitate the digestion and absorption of fat and fat-soluble vitamins in the human digestive system. The tolerance ability among isolated LAB isolates to bile salts was assessed and is shown in Figure 3. Eight isolates including R3, R7, AC1, AC9, AC11.1, AC12, AC13, and AC14.1, could survive in the presence of bile salt concentrations of 0.3% and 0.5%, among which AC1 maintained nearly 100% of relative growth, while *L. plantarum* R7 and *Leu. Mesenteroides* AC11.1 displayed the second highest viability in the MRS supplemented with bile salt concentrations of 0.3% and 0.5%, respectively, compared to the growth in the regular MRS (Figure 3).

**Figure 4:**
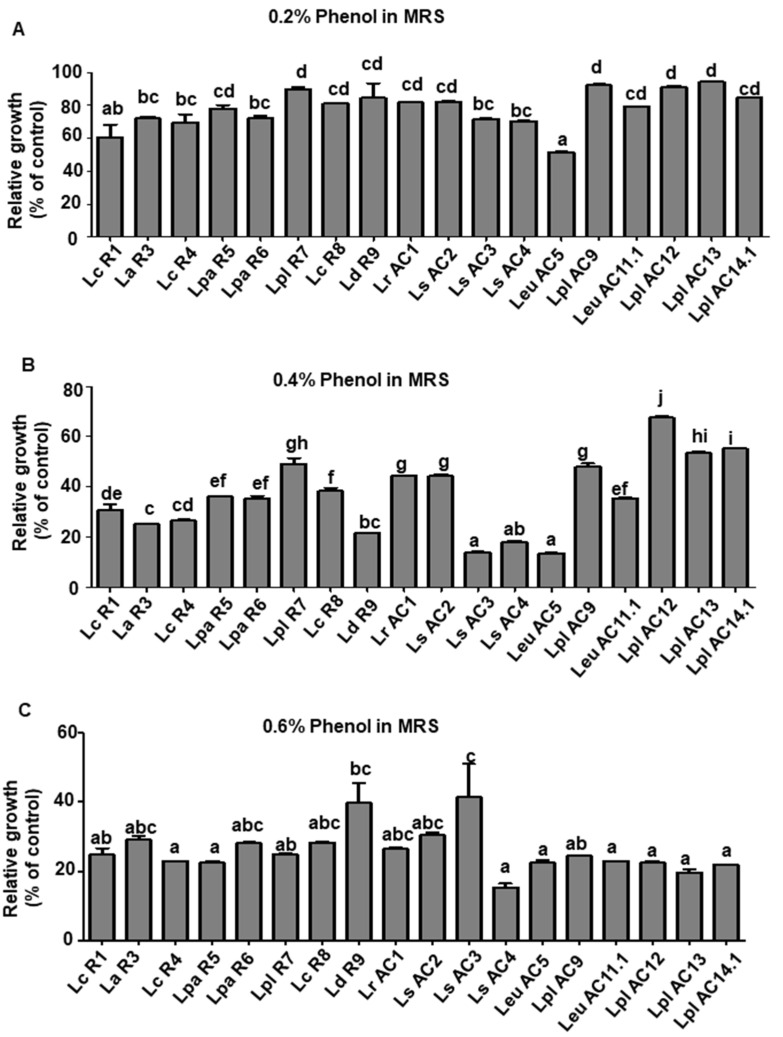
Relative growth of selected LAB isolates in the MRS broth supplemented with 0.2% (**A**), 0.4% (**B**), and 0.6% (**C**) phenol after 24 h of incubation. Relative growth was determined by the ratio of OD_600_ of LAB cultures grown in the MRS containing different concentrations of phenol to that in the regular MRS. Graph bars marked with different letters on top represent statistically significant results. The results were compared by one-way ANOVA and Turkey post hoc test; *p* < 0.05; *n* = 2. Lc, *L. casei*; La, *L. acidophilus*; Lpa, *L. paracasei*; Lpl, *L. plantarum*; Ld, *L. delbrueckii*; Lr, *L. rhamnosus*; Ls, *L. sakei*; Leu, *Leu. mesenteroides*.

In terms of the resistant ability of isolated LAB isolates to phenol, most of the growth curves for the tested LAB reached a plateau after 24 h of incubation regardless of the phenol concentration (Supplementary Figure S3). As shown in Figure 4, all tested LAB isolates showed higher growth in the MRS with lower concentrations of phenol. Compared to the growth at 0 h incubation with 0.2% phenol, the growth of *L. plantarum* (R7, AC9, AC12, and AC13) still showed similar growth after 24 and 48 h of incubation. On the other hand, *Leu. mesenteroides* AC5 exhibited the lowest tolerance ability compared to the other isolates in the presence of 0.2% phenol (Figure 4A). Significant changes were observed in the MRS with 0.4% phenol. *L. plantarum* AC12 had the highest growth level (~75%), while *L. sakei* (AC3 and AC4) and *Leu. mesenteroides* AC5 showed the lowest growth level, which was less than 20% (Figure 4B). The concentration of phenol at 0.6% had a strong inhibitory effect on LAB, as the relative growth of all tested isolates was less than 20% after 24 and 48 h of incubation. Overall, *L. plantarum* (R7, AC12, and AC13) showed higher tolerance to phenol than the other LAB isolates tested in this study.

### 3.2. Antimicrobial Effect of LAB

**Figure 5:**
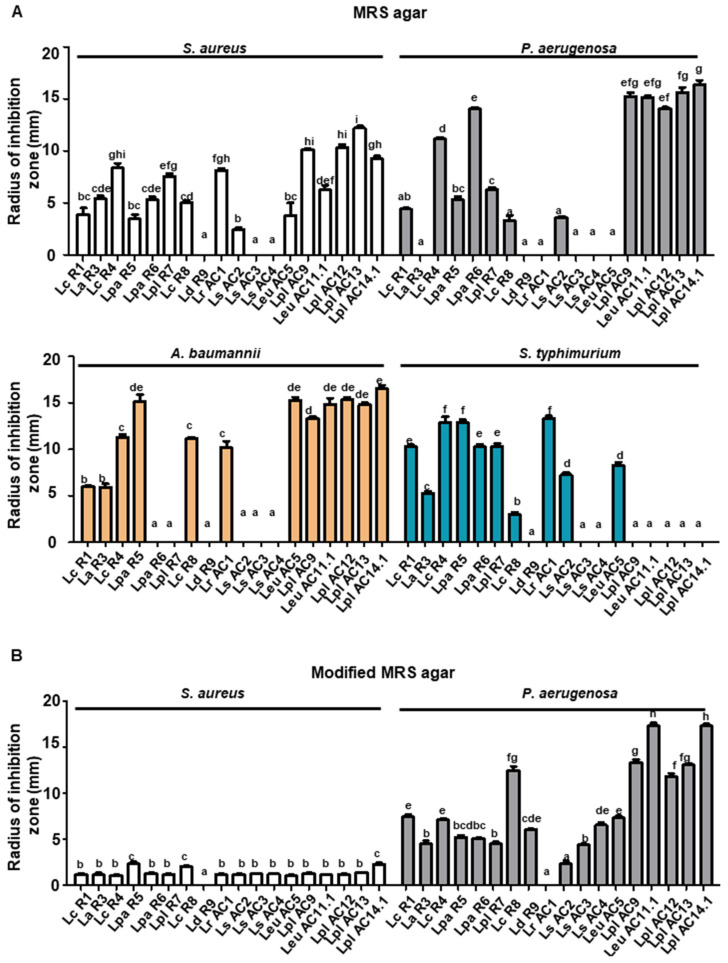
Antimicrobial activity of selected LAB isolates towards different pathogens in MRS agar (**A**) and M-MRS agar (**B**). The radius of inhibition zone was measured in MRS agar plates and modified MRS agar plates. *S. aureus*, *staphylococcus aureus*; *A. baumannii*, *Acinetobacter baumannii*; *Salmonella typhimurium*, *S. typhimurium*; *P. aerugenosa*, *Pseudomonas aerugenosa*. Graph bars marked with different letters on top represent statistically significant results. The results were compared by one-way ANOVA and Turkey post hoc test; *p* < 0.05; *n* = 2. Lc, *L. casei*; La, *L. acidophilus*; Lpa, *L. paracasei*; Lpl, *L. plantarum*; Ld, *L. delbrueckii*; Lr, *L. rhamnosus*; Ls, *L. sakei*; Leu, *Leu. mesenteroides*.

The antimicrobial activities of LAB were determined using regular MRS plates containing 2% glucose, which would be hydrolyzed to produce lactic acid, whereas modified MRS (M-MRS) plates containing 0.2% glucose could indicate that the antibacterial effect, if any, would be due to inhibitory substances other than lactic acid [26]. The inhibition zones of LAB towards *S. aureus* on the MRS plates were generally larger than those on the M-MRS plates, suggesting lactic acid could inhibit the growth of *S. aureus* efficiently (Figure 5). In the plates, not all of the LAB isolates showed regular inhibition zones towards pathogens *S. aureus*, *A. baumannii*, *S. typhimurium*, and *P. aeruginosa*, which were marked with “not detectable (ND)”. *L. casei* (R4), *L. paracasei Shirota* R5, *L. rhamnosus* AC1, and *L. plantarum* (AC9, AC12, and AC13) showed relatively higher antibacterial ability against those pathogens compared to other isolates in regular MRS plates (Figure 5A). By contrast, *L*. *paracasei* R8, *Leu. Mesenteroides* AC11.1, and *L. plantarum* (AC12 and AC13) showed larger inhibition zones in M-MRS than other LAB isolates (Figure 5B).

### 3.3. Adhesive Ability to Caco-2 Cells among LAB Isolates

**Figure 6:**
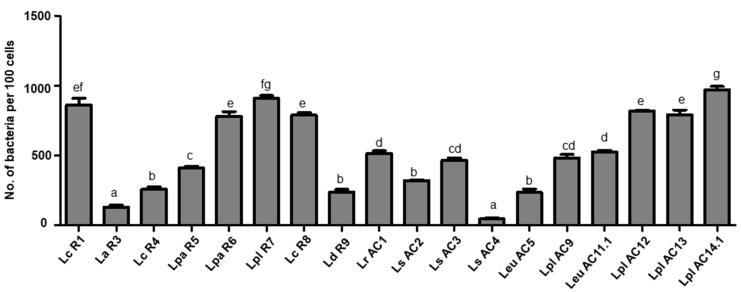
Adhesive ability of selected LAB isolates to Caco-2 cells. Graph bars marked with different letters on top represent statistically significant results. The results were compared by one-way ANOVA and Turkey post hoc test; *p* < 0.05; *n* = 2. Lc, *L. casei*; La, *L. acidophilus*; Lpa, *L. paracasei*; Lpl, *L. plantarum*; Ld, *L. delbrueckii*; Lr, *L. rhamnosus*; Ls, *L. sakei*; Leu, *Leu. mesenteroides*.

The LAB isolates were tested for their ability to adhere to human epithelial cell line Caco-2. The number of bacteria adhered to each 100 Caco-2 cells was calculated (Figure 6). Among the tested isolates, *L. casei* R1 and *L. plantarum* (R7 and AC14.1) exhibited the highest adhesive ability with 863, 910, and 972 per 100 Caco-2 cells, respectively, while *L. acidophilus* R3 and *L. casei* AC4 only adhered to the Caco-2 cells with low numbers of less than 100 bacteria per 100 Caco-2 cells.

### 3.4. Fermented Soymilk by LAB and Its Potential β-Glucosidase Activity

The numbers of live LAB in the soymilk before and after 48 h of fermentation were determined. Except *L. casei* R4 and *L. delbrueckii subsp. Bulgaricus* R9, the other LAB isolates multiplied from the original number of 10^6^ CFU/mL to higher than 10^8^ CFU/mL. After 48 h of fermentation, the numbers of *L. paracasei Shirota* R5, *L. plantarum* R7, *L. rhamnosus* AC1, *L. sakei* AC3, and *Leu. mesenteroides* AC5 increased to more than 10^9^ CFU/mL, suggesting those isolates multiply and survive in the fermentation conditions better than the other isolates during soymilk fermentation (Table 2).

**Figure 7:**
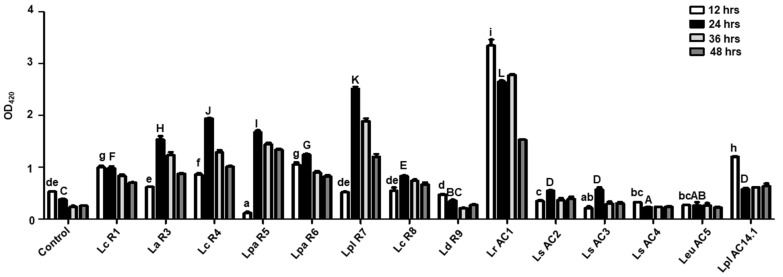
Potential hydrolytic capacity of β-glucosidase in the soymilk fermented by different LAB isolates. The results were compared by one-way ANOVA and Turkey post hoc test; *p* < 0.05; *n* = 2. Lower case letters represent the comparisons of results of groups after 12 h fermentation. Upper case letters represent the comparisons of results of groups after 24 h fermentation. Control: non-fermented soymilk. Lc, *L. casei*; La, *L. acidophilus*; Lpa, *L. paracasei*; Lpl, *L. plantarum*; Ld, *L. delbrueckii*; Lr, *L. rhamnosus*; Ls, *L. sakei*; Leu, *Leu. mesenteroides*.

The β-glucosidase is known to hydrolyze the β-glycosidic bond within the isoflavone structure, converting the glycoside form to the aglycone form, the latter of which is known to be more easily absorbed and beneficial for humans [27]. As the amount of β-glucosidase present in the fermented soymilk was not determined, the rates of ρNPG hydrolysis were measured to compare the overall hydrolytic potential of β-glucosidase present in the same volume of soymilk fermented by different LAB isolates. The β-glucosidase hydrolytic capacity in soymilk fermented by *L. sakei* (AC2, AC3, AC4) and *Leu. mesenteroides* AC5 was much lower than that in the soymilk fermented by other isolates. Overall, the hydrolytic capacity of β-glucosidase was the highest after fermentation for 24 h except AC1 and AC14.1, both of which showed the highest ρNPG hydrolysis rate at 12 h after fermentation and a dropped rate afterwards. Additionally, the soymilk fermented by *L. rhamnosus* AC1 exhibited a higher hydrolytic capacity than those fermented by other isolates (Figure 7). Therefore, AC1-fermented soymilk might contain more aglycone isoflavone than the soymilk fermented by other isolates.

### 3.5. Changes of Soymilk after Fermentation by LAB

**Figure 8:**
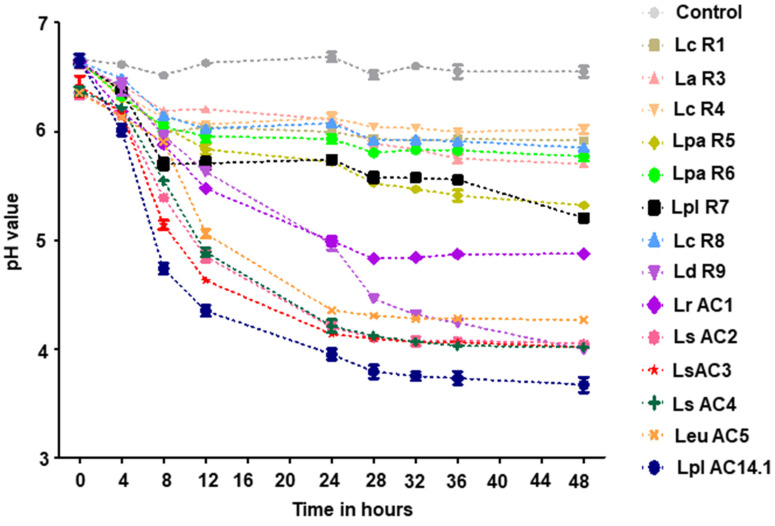
Changes in pH of the soymilk fermented by different LAB isolates. *n* = 2. Lc, L. casei; La, L. acidophilus; Lpa, L. paracasei; Lpl, L. plantarum; Ld, L. delbrueckii; Lr, L. rhamnosus; Ls, L. sakei; Leu, Leu. mesenteroides.

Changes in pH during the soymilk fermentation is a consequence due to lactic acid and possibly other substances produced by LAB during this process. During 48 h of fermentation, the pH of the soymilk fermented by different LAB isolates reduced from an initial pH of 6.4–6.6. The pH values of soymilk fermented by *L. delbrueckii subsp. Bulgaricus* R9, *L. rhamnosus* AC1, *L. sakei* (AC2, AC3, AC4), *Leu. mesenteroides* AC5, and *L. plantarum* AC14.1 decreased to 4.9 or lower compared to the pH of soymilk fermented by the other tested isolates (Figure 8). *L. plantarum* AC14.1 caused the largest reduction in the pH of soymilk among all tested isolates.

**Figure 9:**
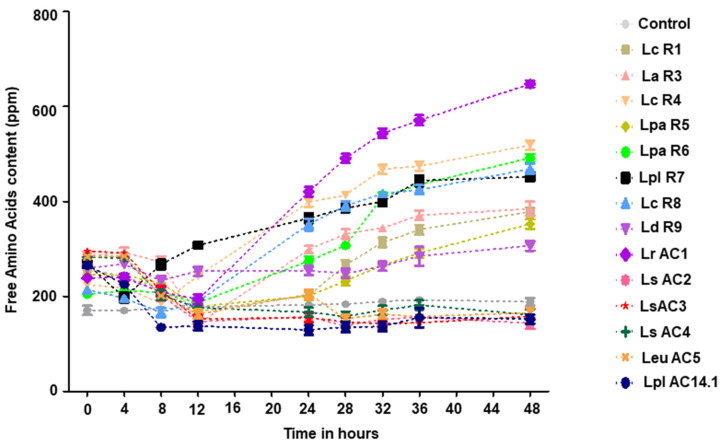
Changes in free amino acid (FAA) content in the soymilk fermented by different LAB isolates at different timepoints during fermentation. *n* = 2. Lc, *L. casei*; La, *L. acidophilus*; Lpa, *L. paracasei*; Lpl, *L. plantarum*; Ld, *L. delbrueckii*; Lr, *L. rhamnosus*; Ls, *L. sakei*; Leu, *Leu. mesenteroides*.

The production of FAA is an important indicator to evaluate the proteolytic ability of LAB in the fermented food (Figure 9). In comparison with non-fermented soymilk, soymilk fermented by *L. casei* (R1, R4), *L. acidophilus* R3, *L. paracasei* (R5, R6, R8), *L. plantarum* R7, *L. delbrueckii subsp. Bulgaricus* R9, and *L. rhamnosus* AC1 exhibited higher FAA content. Among those isolates, *L. rhamnosus* AC1 appeared to possess the highest proteolytic activity among all the isolates tested.

In addition, the content of isoflavones in the nonfermented and fermented soymilk was measured using HPLC. The relative quantity of genistein and daidzein, two major isoflavone aglycones in the fermented soymilk, were determined (Table 3). *L. casei* R1-, *L. plantarum* R7-, *L*. *paracasei* R8-, and and *L. rhamnosus* AC1-fermented soymilk were shown to contain more aglycone forms of isoflavone when compared to other fermented soymilk or non-fermented soymilk (Table 3). Consistent with the hydrolytic potential of β-glucosidase observed in the *L. rhamnosus* AC1-fermented soymilk, high levels of aglycone forms of isoflavone were also observed in this fermented soymilk (Figure 6 and Table 3).

**Figure 10:**
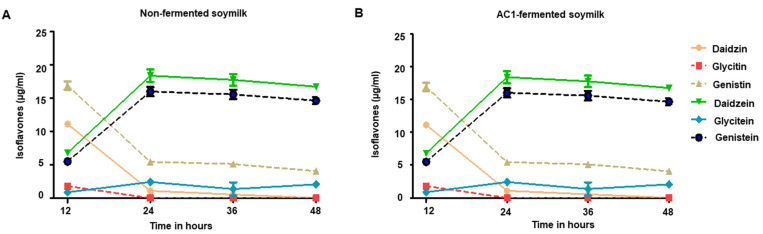
Changes in different forms of isoflavones in the soymilk without (**A**) and with fermentation by AC1 (**B**) at different time points during fermentation. *n* = 2. AC1, *L. rhamnosus* AC1.

To further confirm the changes of main isoflavone forms during the fermentation process of soymilk by *L. rhamnosus* AC1, six major isoflavone forms were measured in the soymilk with and without fermentation by AC1 at different time points during fermentation (Supplementary Table S1). In the non-fermented soymilk, the concentrations of glycoside forms of isoflavone, daidzin, glycitin, and genistin were much higher than those of aglycone forms, daidzein, glycitein, and genistein, up to 36 h of fermentation. It is of interest that the content of daidzin and genistin decreased after 48 h of fermentation in the non-fermented soymilk (Supplementary Table S1 and Figure 10A). The changes in those glycoside forms of isoflavone might be due to long-term incubation in a warm room at 37 °C, which altered the components concentrations. In *L. rhamnosus* AC1-fermented soymilk, after 12 h fermentation, most of the isoflavones were still glycoside forms in *L. rhamnosus* AC1-fermented soymilk. However, after 24 h, almost all the glycoside forms of isoflavone were converted into aglycone forms (Supplementary Table S1 and Figure 10B).

### 3.6. Antioxidant Activities In Vitro

**Figure 11:**
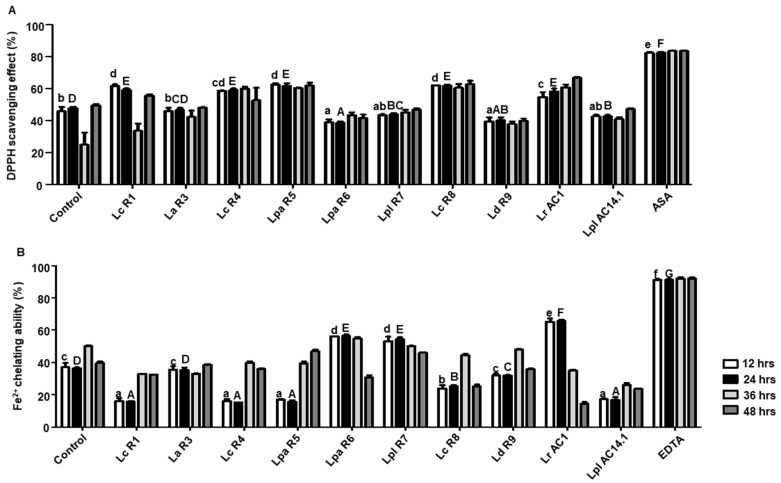
Antioxidant capacities of soymilk fermented by LAB isolates at different time points during fermentation in vitro. Measurement of superoxide anion radical scavenging ability (**A**); Measurement of ferrous ion-chelating ability (**B**). 1 mg/mL of ascorbic acid (ASA) and 0.5 µg/mL of EDTA were used as positive controls for DPPH scavenging effect and Fe^2+^-chelating ability, respectively. Lower case letters represent the comparisons of results of groups after 12 h fermentation. Upper case letters represent the comparisons of results of groups after 24 h fermentation. Lc, *L. casei*; La, *L. acidophilus*; Lpa, *L. paracasei*; Lpl, *L. plantarum*; Ld, *L. delbrueckii*; Lr, *L. rhamnosus*; Ls, *L. sakei*; Leu, *Leu. mesenteroides*.

Isoflavone, especially the aglycone forms of isoflavone, is reported to show high antioxidant ability in vitro. Two approaches, namely the DPPH scavenging ability and ferrous ion-chelating ability, were used to assess the antioxidant ability in this study. In comparison with non-fermented soymilk, the soymilk fermented by *L. casei* (R1, R4), *L. paracasei* (R5, R8), and *L. rhamnosus* AC1 showed higher DPPH scavenging activity than that of the soymilk fermented by other isolates (Figure 11A). However, the soymilk fermented by *L. paracasei* R6, *L. plantarum* R7, and *L. rhamnosus* AC1 exhibited a superior ferrous ion-chelating ability compared to the soymilk fermented by other isolates and non-fermented soymilk (Figure 11B). Overall, the results indicate that *L. rhamnosus* AC1-fermented soymilk possesses the highest antioxidant ability, which is also consistent with the high content of aglycone forms of isoflavone (Supplementary Table S1).

## 4. Discussion

The genera *Bifidobacterium*, *Enterococcus*, *Lactobacillus*, *Lactococcus*, *Leuconostoc*, *Pediococcus*, and *Streptococcus* spp. are LAB that provide health benefits which could be evaluated by some criteria in vitro, including enduring human digestion, gastric juice and bile, and resisting pathogens [16]. Edible probiotic bacteria are firstly attacked by lysozymes in the human mouth, entering the hostile environment of the stomach and reside in the intestine. The pH of the stomach varies depending on the emptiness of the stomach and the presence of different food. In general, the pH of the stomach is around 3.0 in the presence of food even though the pH of hydrochloric acid secreted in stomach is 0.9 [28]. Thus, it is necessary to test the survival ability of LAB isolates in a low pH environment. The bile salts with a detergent function are synthesized from the cholesterol to the primary bile acids in the liver, which are further converted into secondary bile acids by certain intestinal bacteria. Secondary bile salts then undergo enterohepatic circulation within the liver, being stored in the gallbladder, and released into the small intestine during the digestion process. Bile salts could destroy the cell membrane of some bacteria, whose main components are lipids and fatty acids [28]. Some microorganisms, including *Lactobacillus*, can perform the enzymatic deconjugation of bile acids via bile salt hydrolase (BSH), making the unconjugated bile acids precipitate. LAB isolates show different capacities for resisting bile salts, for which producing BSH is one of the possible mechanisms. Phenol tolerance is another important selection criterion, as phenol produced by the intestinal microbiota is a toxic compound in the GI tract. Urease produced by some gut bacteria including *Enterobacteriaceae*, *Peptostreptococcus*, *Clostridia*, and *Eubacteria* hydrolyzes urea into toxic substances such as ammonia, phenol, and indole amine [29]. Other than several *L. plantarum* in our study that showed overall high tolerance to acid, salt, and phenol, *L. rhamnosus* AC1 isolated from baby fecal samples was also able to resist hostile conditions.

As probiotics require a broad antimicrobial spectrum against pathogenic bacteria with strong antimicrobial activity, the antagonistic ability is especially important for those probiotic isolates. The antimicrobial activity of probiotic isolates against pathogens could be attributed to various factors, such as competitive exclusion, immune modulation, as well as production of organic acids, hydrogen peroxide, and antimicrobials [30]. Indeed, probiotics adhering to the intestinal epithelial cells seems to be a prerequisite for the competitive exclusion of enteropathogenic bacteria [31] and immune modulation of the host [32]. The removal of the attached pathogens, such as *Salmonella* and *Clostridia*, from the infected intestinal tract could be stimulated by the adhesive probiotic bacteria *L. rhamnosus* GG [33]. Therefore, successful probiotic bacteria are usually able to colonize the intestine, at least temporarily, by adhering to the intestinal mucosa. Several isolates belonging to *L. plantarum* and *L. rhamnosus* among those isolated in this study exhibited strong antimicrobial activity. Within the tested isolates in this study, *L. rhamnosus* AC1 showed an average adhesion ability with 514 CFU per 100 Caco-2 cells.

Except evaluating LAB isolates in the hostile conditions, the characteristics of soymilk fermented by those isolates were also determined. Enzymatic degradation of proteins is an important process in the fermentation of foods containing a high protein content [34]. In addition, proteolysis mainly contributes to product attributes including texture, appearance, as well as characteristic aroma and taste of fermented products. Dry soybean contains approximate 36% protein, which, when undergoing metabolization by probiotics, yields bioactive peptides known to confer health benefits [35].The proteolytic activity of probiotic isolates could cleave the soy protein into various amino acids and peptides. Our results show that *L. rhamnosus* AC1-fermented soymilk contains higher free amino acids than soymilk fermented by other isolates, implying its excellent potential to contain more bioactive peptides, which requires further investigation.

Other than the soy peptides, another considerable important category of substances harboring beneficial effects on human health is soy isoflavones. It is reported that isoflavones are readily absorbed, metabolized, and excreted, although there are individual and sex-related differences [36]. Isoflavones have two major forms: the aglycones and glycosides [21]. Since the structure itself is a limiting factor for the absorption from gastrointestinal tract, the chemical forms of aglycones are more readily absorbed and more bioavailable than glycosides [21]. During the process of digestion, the acetyl and malonyl derivatives of genistin and daidzin are metabolized to become genistin and daidzin, which are then hydrolyzed in the large intestine by bacteria, resulting in the removal of sugar moiety to produce their respective aglycones, daidzein and genistein, respectively [37]. Previous studies have shown that the glycoside forms must be first hydrolyzed into aglycone forms by β-glucosidases of gut microflora to be absorbed in vivo [38]. This knowledge has led to the development of aglycon-enriched products obtained from soybean products treated with β-glucosidase directly or fermentation with LAB [39]. The β-glucosidase hydrolytic efficiency in the *L. rhamnosus* AC1-fermented soymilk was the highest in comparison with the same volume of soymilk fermented by other isolates. Consistently, the content of isoflavone aglycone forms was the highest in the *L. rhamnosus*-fermented soymilk compared to the same volume of soymilk fermented by other isolates tested in this study.

Another crucial feature of isoflavones is their antioxidant ability. It is generally believed that the beneficial effects of isoflavones might be related to their antioxidant activity. Aglycones, especially genistein and daidzein, have greater antioxidant activities than their glycoside forms [40]. Genistein is able to suppress microsomal lipid peroxidation induced by the Fe^2+^-ADP complex and NADPH [41], inhibit NADH oxidase and the respiratory chain in rat liver mitochondria [42], and target mitogen-activated protein to deregulate cellular response [43]. Our data show that *L. rhamnosus* AC1-fermented soymilk possesses the highest antioxidant activity in vitro, which is consistent with its the high levels of aglycones.

By assessing all the isolates tested in this study, we conclude that the isolate *L. rhamnosus* AC1, isolated from infant feces, showed relatively better tolerant abilities and has the best fermentation characteristics in soymilk, implying that it may have greater potential than the other LAB isolates tested in future studies.

## 5. Conclusions

The characteristics of eighteen isolates were evaluated by different testing approaches. Several isolates belonging to *L. plantarum* and *L. rhamnosus* showed high tolerance. By contrast, the isolates of *L. plantarum* and *L. paracasei* exhibited a greater adhesive ability than the other test isolates. As LAB isolates are able to alter the unfavorable beany flavor of soymilk and increase its nutritional value, the characteristics of soymilk fermented by those probiotic isolates were also evaluated. Overall, *L. rhamnosus* AC1 exhibited the best performance in the fermented soymilk among all the tested isolates. It is of importance that *L. rhamnosus* AC1-fermented soymilk contained the highest activity of β-glucosidase, which could effectively convert isoflavones from glycosides into aglycones. In addition, the highest antioxidant activities including DPPH scavenging ability and iron-chelating ability were also reported in the *L. rhamnosus*-fermented soymilk among the soymilk fermented by other isolates. Altogether, *L. rhamnosus* AC1, which showed relatively better characteristics in the simulated gastric and intestinal conditions and the best performance in fermented soymilk, is an isolate that has potential for further investigation of its potential health benefits for humans.

## Figures and Tables

**Table 1:** Description of the lactic acid bacteria cultures used in this study.

Label	Isolates in This Study	Source
Lc R1	*L. casei*	HKU
La R3	*L. acidophilus*, *NCFM*	ATCC
Lc R4	*L. casei*, *NBRC15883*	Cheese
Lpa R5	*L. paracasei Shirota*	Yakult drink
Lpa R6	*L. paracasei*	HKU
Lpl R7	*L. plantarum*, *BCRC910734*	Kimchi
Lc R8	*L. casei/L. paracasei*	Yoghurt
Ld R9	*L. delbrueckii subsp. Bulgaricus*	Yoghurt
Lr AC1	*L. rhamnosus*	Infant feces
Ls AC2	*L. sakei*	Jinmi Korean kimchi
Ls AC3	*L. sakei*	Seoul farm Korean kimchi
Ls AC4	*L. sakei*	Select Korean kimchi
Leu AC5	*Leu. mesenteroides*	Select Korean kimchi
Lpl AC9	*L. plantarum*	kombucha
Leu AC11.1	*Leu. mesenteroides*	Taiwan kimchi
Lpl AC12	*L. plantarum*	Taiwan kimchi
Lpl AC13	*L. plantarum*	Taiwan kimchi
Lpl AC14.1	*L. plantarum*	Taiwan kimchi

HKU: The University of Hong Kong; ATCC: American Type Culture Collection.

**Table 2:** Viable counts of fermented soymilk before and after fermentation by selected LAB isolates.

LAB Isolates	Viable Counts (log CFU/mL)	
	Before Fermentation	After Fermentation
Control	0.00 ± 0.00 ^a^	0.00 ± 0.00 ^a^
Lc R1	6.55 ± 0.01 ^d^	8.41 ± 0.05 ^ab^
La R3	6.11 ± 0.01 ^abc^	8.60 ± 0.02 ^abc^
Lc R4	6.49 ± 0.14 ^d^	7.12 ± 0.09 ^ab^
Lpa R5	6.50 ± 0.02 ^d^	9.29 ± 0.14 ^d^
Lpa R6	6.24 ± 0.02 ^c^	8.89 ± 0.02 ^abcd^
Lpl R7	6.14 ± 0.00 ^bc^	9.20 ± 0.19 ^cd^
Lc R8	5.92 ± 0.06 ^abc^	8.36 ± 0.10 ^ab^
Ld R9	5.23 ± 0.12 ^ab^	7.59 ± 0.08 ^ab^
Lr AC1	6.09 ± 0.04 ^abc^	9.07 ± 0.06 ^abcd^
Ls AC2	6.49 ± 0.02 ^abc^	8.42 ± 0.00 ^abc^
Ls AC3	6.15 ± 0.02 ^bc^	9.04 ± 0.01 ^abcd^
Ls AC4	6.20 ± 0.03 ^c^	8.25 ± 0.03 ^b^
Leu AC5	6.23 ± 0.00 ^c^	9.07 ± 0.06 ^bcd^
Lpl AC14.1	6.49 ± 0.02 ^d^	8.69 ± 0.05 ^abc^

Fermentation was carried out at 37 °C for 48 h. Control, non-fermented soymilk. Different letters in the same column represent statistically significant results. The results were compared by one-way ANOVA and Turkey post hoc test within columns; *p* < 0.05; *n* = 2. Lc, *L. casei*; La, *L. acidophilus*; Lpa, *L. paracasei*; Lpl, *L. plantarum*; Ld, *L. delbrueckii*; Lr, *L. rhamnosus*; Ls, *L. sakei*; Leu, *Leu. mesenteroides.*

**Table 3:** Content of main isoflavone forms, genistein and daidzein, in the soymilk fermented by LAB isolates after 48 h.

Samples	Peak Area (mAu)	
	Genistein	Daidzein
100 ppm Genistein	14,368.00 ± 401.64 ^c^	ND
100 ppm Daidzein	ND	12,354.50 ± 456.08 ^c^
Control	911.00 ± 14.14 ^b^	843.50 ± 20.51 ^b^
Lc R1	1003.50 ± 43.12 ^b^	875.00 ± 11.31 ^b^
Lc R4	858.50 ± 45.96 ^b^	875.00 ± 11.31 ^b^
Lpa R5	819.50 ± 12.02 ^b^	745.00 ± 8.49 ^b^
Lpa R6	831.00 ± 2.83 ^b^	741.00 ± 16.97 ^b^
Lpl R7	949.50 ± 10.61 ^b^	843.50 ± 20.51 ^b^
Lc R8	1023.50 ± 21.92 ^b.^	826.00 ± 14.14 ^b^
Ld R9	210.50 ± 6.36 ^a^	144.00 ± 9.90 ^a^
Lr AC1	1041.50 ± 13.44 ^b^	866.50 ± 14.85 ^b^
Ls AC2	148.00 ± 12.73 ^a^	134.50 ± 7.78 ^a^
Ls AC3	156.00 ± 8.49 ^a^	135.50 ± 14.85 ^a^
Lpl AC14.1	845.50 ± 13.44 ^b^	746.00 ± 9.90 ^b^

ND: not detectable. Different letters in the same column represent statistically significant results. The results were compared by one-way ANOVA and Turkey post hoc test within columns; *p* < 0.05; *n* = 2. Lc, *L. casei*; La, *L. acidophilus*; Lpa, *L. paracasei*; Lpl, *L. plantarum*; Ld, *L. delbrueckii*; Lr, *L. rhamnosus*; Ls, *L. sakei*; Leu, *Leu. mesenteroides.*

## Data Availability

Not applicable.

## Supplementary Materials

The following are available online at https://www.mdpi.com/article/10.3390/microorganisms9122544/s1, Figure S1: Tolerance ability to acid of LAB in regular MRS broth and MRS of pH 2.0, 3.0 and 4.0, Figure S2: Tolerance ability of LAB to 3% NaCl, 6% NaCl and 9% NaCl in MRS, Figure S3: Tolerance ability of LAB to 0.2%, 0.4%, and 0.6% phenol in MRS, Table S1: Changes of different isoflavone forms in non-fermented soymilk and *L. rhamnosus* (AC1)-fermented soymilk at different time points during fermentation.
