# Supplementary material for: Characterization of the Probiotic Potential of Lactic Acid Bacteria Isolated from Kimchi, Yogurt, and Baby Feces in Hong Kong and Their Performance in Soymilk Fermentation
Source: Microorganisms. 2021 Dec 9;9(12):2544. doi: 10.3390/microorganisms9122544 (PMC8705588; doi:10.3390/microorganisms9122544)
Supplement: Supplementary file 1 [file microorganisms-09-02544-s001.zip › microorganisms-1483528-supplementary.pdf]

Supplementary results

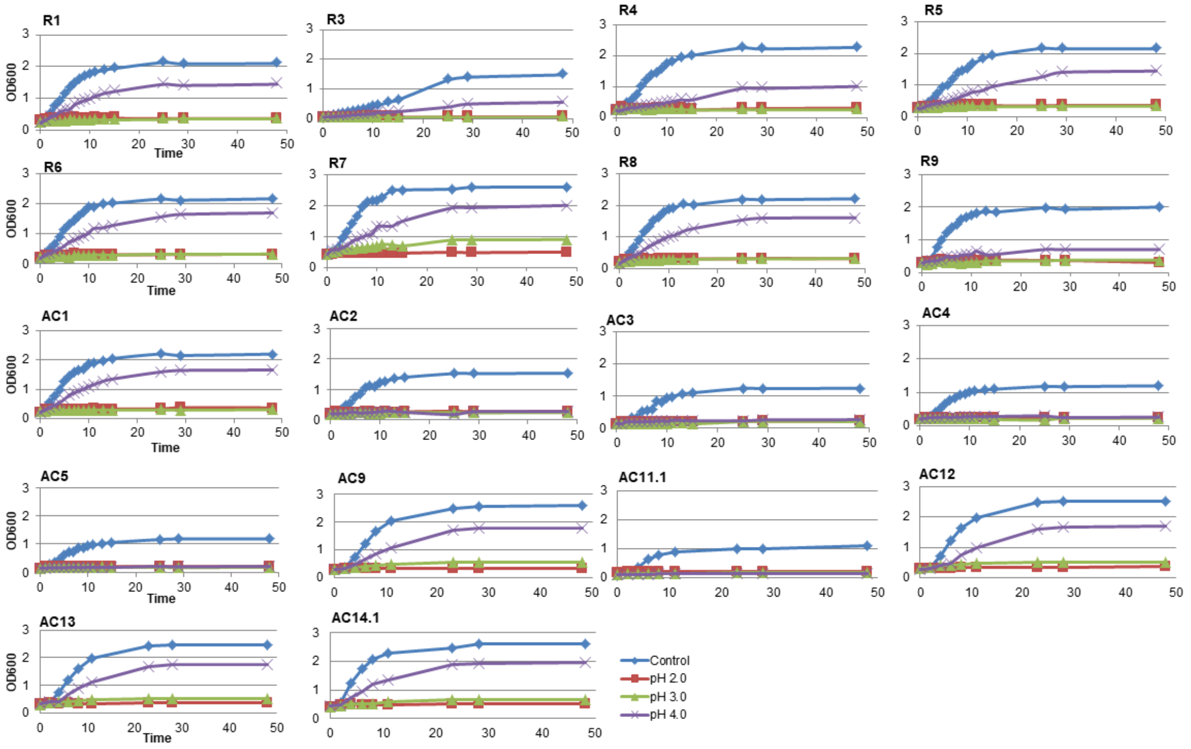

Sup. Figure S1. Tolerance ability of selected LAB strains to acid, pH 2.0, 3.0 and 4.0 MRS broth.

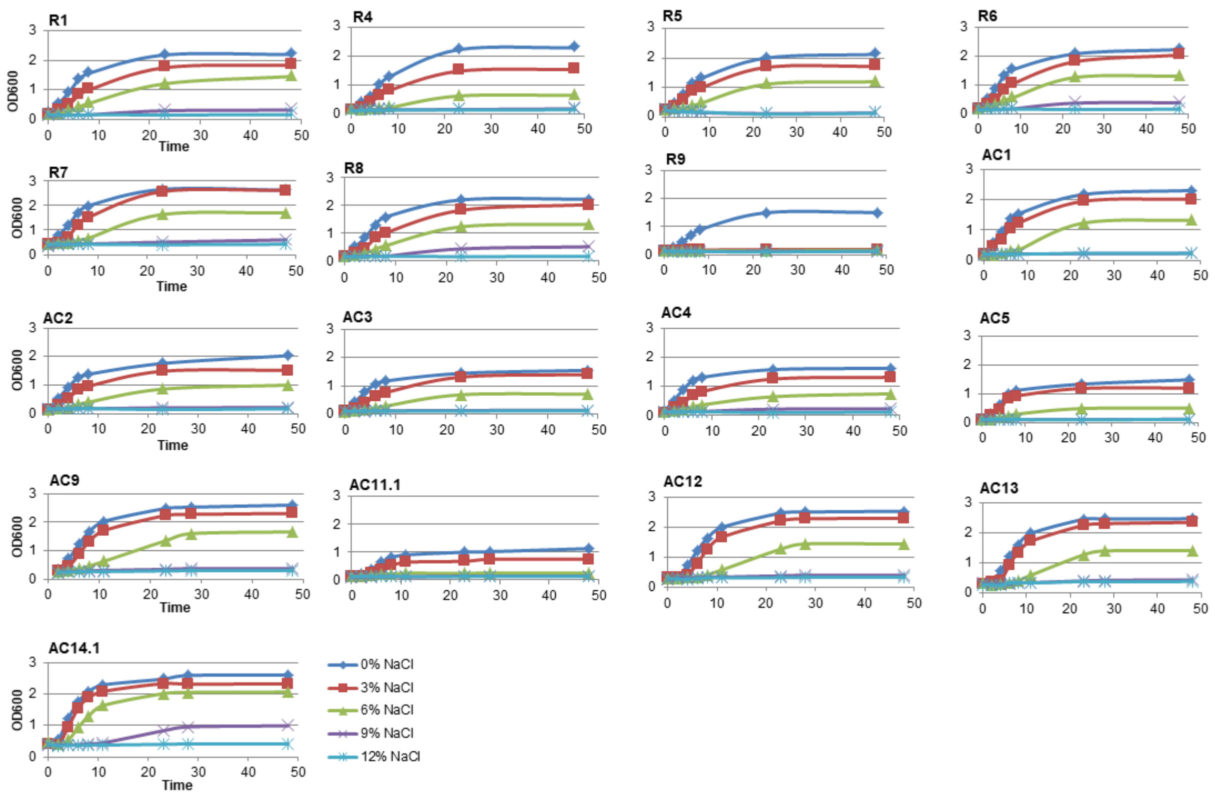

Sup. Figure S2. Tolerance ability of selected LAB strains to 3% NaCl, 6% NaCl and 9% NaCl in MRS.

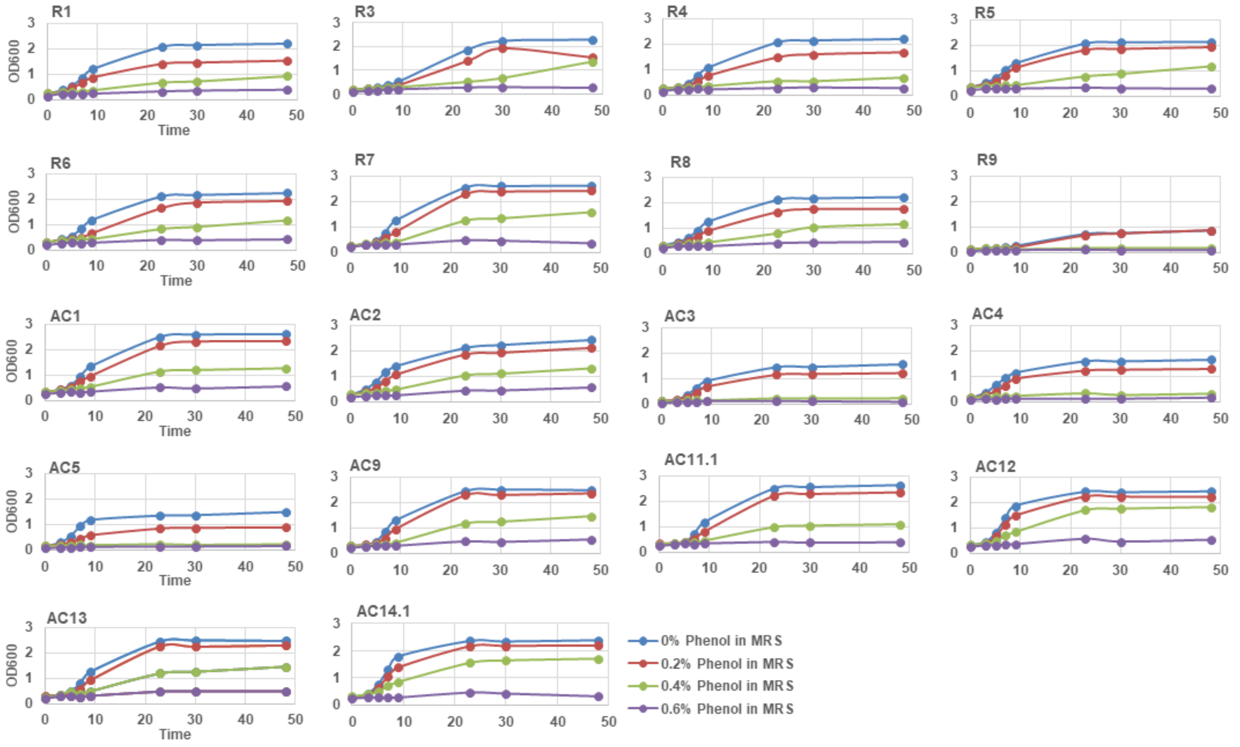

Sup. Figure S3. Tolerance ability of selected LAB strains to 0.2%, 0.4%, and 0.6% phenol in MRS.

Sup. Table S1. Changes of different isoflavone forms in non-fermented soymilk and *L. rhamnosus* AC1-fermented soymilk at different time points during fermentation.

Isoflavone forms	Control (non-fermented soymilk)				AC1-fermented soymilk			
	12 hrs	24 hrs	36 hrs	48 hrs	12 hrs	24 hrs	36 hrs	48 hrs
Daidzin	11.39 ± 1.42^{cd}	13.38 ± 0.89^{d}	13.06 ± 0.24^{cd}	7.18 ± 0.18^{b}	11.13 ± 0.37^{c}	1.10 ± 0.09^{a}	0.55 ± 0.00^{a}	ND
Glycitin	2.87 ± 1.18^{c}	2.37 ± 0.43^{bc}	1.99 ± 0.03^{bc}	1.28 ± 0.04^{ab}	1.80 ± 0.04^{bc}	ND	ND	ND
Genistin	16.14 ± 1.51^{c}	19.78 ± 1.16^{d}	19.47 ± 0.44^{d}	11.11 ± 0.32^{b}	16.92 ± 0.62^{c}	5.44 ± 0.24^{a}	5.10 ± 0.22^{a}	4.05 ± 0.15^{a}
Daidzein	5.15 ± 1.00^{a}	5.86 ± 0.57^{a}	5.33 ± 0.09^{a}	8.74 ± 0.38^{b}	6.81 ± 0.23^{ab}	18.41 ± 0.92^{c}	17.78 ± 0.87^{c}	16.75 ± 0.45^{c}
Glycitein	1.55 ± 0.78^{a}	1.11 ± 0.37^{a}	0.73 ± 0.02^{a}	1.13 ± 0.02^{a}	0.87 ± 0.07^{a}	2.43 ± 0.28^{a}	1.36 ± 1.00^{a}	2.07 ± 0.06^{a}
Genistein	3.47 ± 0.86^{a}	3.95 ± 0.58^{ab}	3.50 ± 0.14^{a}	6.78 ± 0.34^{c}	5.52 ± 0.15^{bc}	16.02 ± 0.69^{d}	15.60 ± 0.71^{d}	14.66 ± 0.53^{d}

Daidzin, Glycitin and Genistin are glycoside forms of isoflavone while Daidzein, Glycitein, and Genistein are aglycone forms of isoflavone. The results were compared by one-way ANOVA and Turkey post hoc test within rows; $p < 0.05$; n = 2.
